# RBFNN-PSO Intelligent Synchronisation Method for Sprott B Chaotic Systems with External Noise and Its Application in an Image Encryption System

**DOI:** 10.3390/e26100855

**Published:** 2024-10-10

**Authors:** Yanpeng Zhang, Jian Zeng, Wenhao Yan, Qun Ding

**Affiliations:** 1 Electrical Engineering College, Heilongjiang University, Harbin 150080, China; zyp_shxy@163.com (Y.Z.); 2211790@s.hlju.edu.cn (J.Z.); wenhaoyan@aliyun.com (W.Y.); 2 Suihua University Artificial Intelligence Innovation and Application Engineering Technology Research Centre, Suihua University, Suihua 151100, China

**Keywords:** Sprott B system, RBFNN-PSO synchronisation method, Zigzag disruption method

## Abstract

In the past two decades, research in the field of chaotic synchronization has attracted extensive attention from scholars, and at the same time, more synchronization methods, such as chaotic master–slave synchronization, projection synchronization, sliding film synchronization, fractional-order synchronization and so on, have been proposed and applied to chaotic secure communication. In this paper, based on radial basis function neural network theory and the particle swarm optimisation algorithm, the RBFNN-PSO synchronisation method is proposed for the Sprott B chaotic system with external noise. The RBFNN controller is constructed, and its parameters are used as the particle swarm particle optimisation parameters, and the optimal values of the controller parameters are obtained by the PSO training method, which overcomes the influence of external noise and achieves the synchronisation of the master–slave system. Then, it is shown by numerical simulation and analysis that the scheme has a good performance against external noise. Because the Sprott B system has multiple chaotic attractors with richer dynamics, the synchronization system based on Sprott B chaos is applied to the image encryption system. In particular, the Zigzag disambiguation method for top corner rotation and RGB channel selection is proposed, and the master–slave chaotic system synchronisation sequences are diffused to the disambiguated data streams, respectively. Therefore, the encryption and decryption of image transmission are implemented and the numerical simulation results are given, the random distribution characteristics of encrypted images are analysed using histogram and Shannon entropy methods, and the final results achieve the expected results.

## 1. Introduction

Chaos theory is a popular research topic in nonlinear systems theory. A chaotic motion state is a seemingly random motion that occurs in a dynamical system, which in essence is the sensitivity of the long-term behaviour of the system to the initial conditions [1,2,3]. Chaotic systems are also characterised by unpredictability, non-periodicity, natural iteration and pseudo-randomness, and these properties have led to a wide range of applications in fields such as cryptography, secure communications, signal processing and image encryption [4,5,6,7,8]. In 1990, the scholars Pecora and Carrol proposed the chaotic self-synchronisation method, in which synchronisation between two chaotic systems was achieved for the first time by the drive–response method, while the first observation of the chaotic synchronisation phenomenon was made on electronic circuits [9,10]. This groundbreaking research progress breaks the traditional notion that chaotic motion patterns are uncontrollable and dangerous. It shows that chaos is not only controllable and synchronisable, but that it also can be used as one of the components of the fundamental theoretical study of the dynamics of information transmission and processing, thus making it possible to apply chaotic systems to the field of confidential information communication.

It is well known that external noise is prevalent in real communication processes. The presence of noise not only increases the challenge of chaotic synchronisation, but also easily destroys the established synchronisation state. Therefore, the design of chaotic synchronisation controllers in the presence of external noise must be investigated [11]. In Ref. [12], Cheng propose a synchronisation scheme based on a chaotic system with bounded noise and uncertain parameters to achieve the asymptotic synchronisation of two different chaotic systems, on the basis of the theory of Lyapunov stability and the deadband algorithm. In Ref. [13], Yang et al. design a reduced-order observer asymptotically to estimate the state of the enhanced auxiliary drive system and propose a method that can recover the information signal as well as reconstruct the signal recovery method with uncertain parameter interference and channel noise. In Ref. [14], Xu et al. design a decay function that could decay to zero in a finite time to accelerate the convergence of the global sliding mode surface and construct an improved Global Nonlinear Integral Sliding Mode Squares (GNISMS) to achieve the synchronous control of chaotic systems with external disturbances and internal parameter uncertainties.

Multi-attractor chaotic systems have been emphasized by scholars due to their richer dynamical behaviours. Multi-attractor is a phenomenon in which a chaotic system produces the coexistence of multiple attractors when the system parameters are the same. In Ref. [15], Sprott studies general three-dimensional autonomous constant differential systems with second-order nonlinearities, revealing 19 different examples of chaotic flows, named in the order of the letters A-S, of which the Sprott B system is one. In Ref. [16], the scholars Carroll and Pecora conduct a study of symmetric chaotic systems by synchronizing two multi-attractor Rössler systems with a unidirectional drive, proving that the response system is always synchronized with the drive system, even though it has two attractors, thus demonstrating that multi-attractor chaotic systems can be used in confidential communication systems. In Ref. [17], the problem of the coexistence of multiple attractors in a Sprott B system is investigated, and an extension of the Sprott B system with a polynomial function is proposed to enable the system to generate several different independent attractors, which enhances the dynamical behaviour of the system. In Ref. [18], Ramesh et al. propose a 4D memory mixing degree Sprott B system that adjusts the symmetry of the proposed model through a deviation term, which in turn induces the homogeneous and heterogeneous behaviours of the chaotic system. Based on the needs of image encryption system design, this paper uses the polynomial extended Sprott B system proposed by Lai et al. in Ref. [15] to perform synchronization control research.

At present, researchers have designed a large number of intelligent synchronization control schemes using the rapid development of chaos control theory, intelligent neural controllers and hardware computing resources. In Ref. [19], the information-granularity-based fuzzy radial basis function neural network (IG-FRBFNN) is used to dynamically obtain the weights of each source image, chaotic brainstorm optimization (CBSO) is finally proposed, and the structure optimization and parameter optimization of the network are carried out, respectively. In Ref. [20], a flow inference measurement method based on improved radial basis function neural network (RBFNN) is used to solve the problem of the inaccurate estimation of the valve flow rate, which is affected by the nonlinearities and uncertainties of multiple parameters, and particle swarm optimization (PSO) combined with a least squares algorithm is used to optimize the parameters of the RBFNN, such as the centre, the width and the weights. In Ref. [21], the classical variable projection algorithm (VP) is applied to radial basis function neural networks, and the problem corresponding to the sum of squares of errors (SSE) of the minimization of the general radial basis function neural network (GRBFNN) is transformed into a lower dimensional optimization problem, and it is theoretically proved that the smooth point set of the objective function of the lower dimensional problem is equivalent to that of the original objective function. In Ref. [22], a black-box and Multi-objective Particle Swarm Optimization (MOPSO) algorithm is proposed to optimize the parameters of RBFNN to achieve the prediction of the performance of a cyclone with variable pitch spiral baffle when the cyclone cone becomes rough. The analysis shows that the RBFNN, as a controller, is able to approximate nonlinear functions with arbitrary accuracy and has good generalization ability.

Optimization algorithms play an important role in the parameter optimization of neural network controllers, and Genetic Algorithms (GAs), Gray Wolf Optimization Algorithms (GWOs), Particle Swarm Optimization Algorithms (PSOs), etc., have been widely used in various optimization problems. In Ref. [23], a steganography technique based on a general hybrid chaotic mapping of the 3D displacement function of pixels of a colour image is proposed and a genetic algorithm with permutation coding is proposed to optimize the parameters of the 3D displacement function, which improves the transparency and security of this steganography technique. A new local stochastic optimization strategy is proposed in Ref. [24] to compensate for the defects of GWOs. The method randomly selects several points near the current position of each individual as candidate points in the axial direction and chooses the best point to participate in the update decision of the individual. In Ref. [25], the problems of premature convergence and the lower convergence accuracy of the gray wolf optimization algorithm are addressed, and an improved scale-free network topology (SFGWO)-based gray wolf optimization algorithm is proposed to solve these problems. The improved algorithm first adopts a population construction strategy based on a scale-free network topology, in which the interactions between particles are limited to the topological neighbour directions, which helps to improve the exploration ability of the algorithm. Compared with the gray wolf optimization algorithm, the particle swarm algorithm also has the global search capability, in which the global optimal solution is found through the continuous iteration of particles in the solution space. In Ref. [26], a chaotic dynamic weights particle swarm optimization (CDW-PSO) algorithm is proposed; this introduces chaotic mapping and dynamic weights to modify the search process, and overcomes the problems of the PSO algorithm, such as premature convergence and its easy capture of the local optimal solution. In Ref. [27] applies the PSO optimization algorithm to cluster routing design and proposes an improved cluster routing protocol based on levy chaotic particle swarm optimization (LCPSO-CRP), which enhances the routing efficiency of sensor networks. Based on the above, this paper adopts PSO as the optimization algorithm for the RBFNN controller parameters, and in order to overcome the problem of the PSO algorithm being prone to falling into the local optimal solution, it adopts the method of linear dynamic change of weights from the literature [26], which enhances the global searching ability of the PSO algorithm solution space.

In summary, in this paper, based on the theory of the radial basis function neural network (RBFNN) and particle swarm training algorithm (PSO), we propose a synchronous design scheme for the externally noisy chaotic system RBFNN-PSO, and the main contributions are as follows:

1. A controller based on RBFNN is constructed, considering the influence of external noise on the performance of the system, and the controller parameters are optimized by the group intelligent optimization algorithm to realize the suppression of the influence of external noise on the performance of the synchronous system.
2. The PSO optimization algorithm is selected and improved, and the linear dynamic adjustment of the optimization parameters is used to update the weights, so as to overcome the problem of the PSO optimization algorithm being prone to falling into the local optimal situation; meanwhile, the characteristics of the rich dynamics behaviour of the multi-attractor chaotic system are taken into account, the Sprott B system is selected as the synchronization object, and the parameters of the synchronization controller of the Sprott B chaotic system are introduced into the training process, so as to achieve the optimal solution of the controller parameters of the chaotic system, which is the best solution for the chaotic system. The optimal solution of the controller parameters realizes the consistent synchronization of the two Sprott B systems.
3. In order to enhance the complexity of image encryption, an improved Zigzag top-angle rotation image disruption algorithm is proposed to overcome the problem of the first data in the first place not changing and being easily recognized when the Zigzag is disrupted; at the same time, the method in which the mean size of the image determines the disruption channel is adopted to expand the encrypted secret key space; an analysis of the security performance of the image encryption is carried out, and the encryption system has a better resistance to the differential attack and statistical analysis.

The remaining parts of this paper are organised as follows: Section 2 mainly introduces the basic knowledge of RBF neural networks and particle swarm algorithms, explains the problems solved in this thesis, and constructs the master–slave synchronisation system as an example of the Sprott B system; Section 3 mainly introduces the Sprott B chaotic synchronisation scheme with the specific PSO training process, gives the optimal values of the controller parameters, and analyses the system performance, the overall scheme of image encryption and the enhanced Zigzag disruption algorithm and diffusion method. The simulation results are presented along with the safety analysis in Section 4; Section 5 gives a summary of the whole paper.

## 2. Proposed Model and Preliminary Work

### 2.1. RBF Neural Network

**Figure 1:**
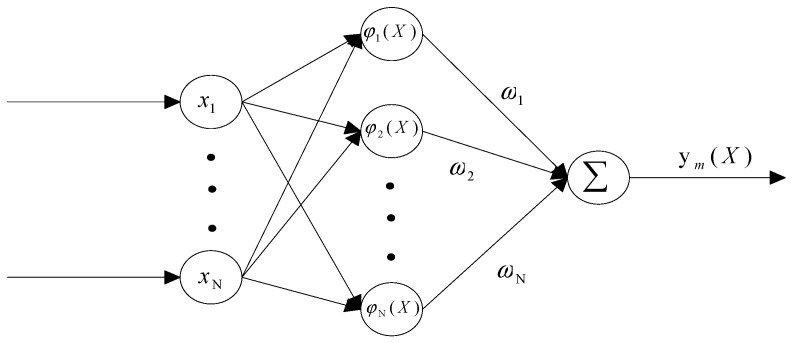
Block diagram of the structure of the radial basis function neural network.

RBF neural networks are widely used in many fields such as time series prediction, classification, function approximation and system control [28,29]. In the current study, the RBF neural network can estimate any disturbance and uncertainty, then estimate its output, which in turn can be used to find the optimal solution to the uncertain parameters of the chaotic system. There can be many form of RBF; in this paper, the Gaussian function is chosen as the interpolation basis function [30]. Thus, the output of the RBF neural network is calculated as follows:

(1) $$y_{m}(X)=\sum_{k=1}^{K}w_{km}\phi_{k}(X)-\theta_{0m},\;m=1,2,\cdots ,M$$

(2) $$\phi_{k}(X)=\exp(-\frac{{\left(X-C_{k}\right)}^{2}}{2\delta_{k}^{2}}),\;k=1,2,\cdots ,K$$

where $w_{km}$ is the ideal constant weight value of the radial basis function, K is denoted as the number of nodes in the hidden layer, and M denotes the number of outputs of the radial basis function; X is denoted as the *N*-dimensional input vector and its individual components are $x_{i}(i=1,2,\cdots ,N)$, and is also denoted as the *N*-dimensional vector, which is the centroid position vector of $\phi_{k}(X)$, with the individual components of $\phi_{k}(X)$ being $C_{jk}(j=1,2,\cdots ,N)$; and $\delta_{k}$ is denoted as the value of the width of the radial basis function, which is also one of the parameters being trained. The structure of the radial basis function neural network is shown in Figure 1.

### 2.2. The PSO Algorithm

The Particle Swarm Algorithm (PSO) was co-proposed by Eberhart and Kennedy [31]. The PSO algorithm process is a population-based search process, in which individuals are called particles, and their positions (states) change successively with time. In a PSO system, particles fly around in a multidimensional search space, and during the flight, every other particle adjusts its position based on its own experience and the neighbouring particles, and then adjusts to the optimal position by using its own positional information and that of the neighbouring particles [32]. In PSO, each particle has a velocity and a position as follows:

(3) $$v_{i}(k+1)=\varphi_{i}(k)v_{i}(k)+\alpha_{1}\gamma_{1i}(P_{i}-x_{i}(k))+\alpha_{2}\gamma_{2i}(G-x_{i}(k))$$

(4) $$x_{i}(k+1)=x_{i}(k)+v_{i}(k+1)$$

where i is denoted as the particle index, k is the discrete time index, and $v_{i}$ is the velocity of the *i*-th particle; $x_{i}$ is the current position of the *i*-th particle and $P_{i}$ is the personal best of the *i*-th particle; G is denoted as the global best of the group, and $\gamma_{1i}$ and $\gamma_{2i}$ are the random numbers on the interval [0, 1] that correspond to the *i*-th particle; $\varphi_{i}(k)$ is denoted as the inertia function; and $\alpha_{1}$ and $\alpha_{2}$ are acceleration constants, which generally take values in [0, 2].

### 2.3. Design Methodology for Synchronous System Controllers

In this paper, the synchronisation of master–slave Sprott B chaotic systems with additional additive noise is discussed. The concept of synchronisation means that after a period of time, the state trajectories of the slave chaotic system gradually converge with those of the master chaotic system, and finally an asymptotically stable state is achieved. For this purpose, we define the master chaotic system and the slave chaotic system as Equations (5) and (6), respectively [11].

(5) $${\dot{x}}^{m}=f(x^{m})+g(x^{m})$$

(6) $${\dot{x}}^{s}=f(x^{s})+g(x^{s})+\varphi (x^{s})+u$$

where $x^{m},\;x^{s}\in R^{n}$, which denote the state vectors of the master and slave systems, respectively. f(⋅), g(⋅) are denoted as linear and nonlinear continuously differentiable functions, respectively. φ(⋅) denotes bounded additional additive noise, and u is denoted as the controller.

There are a number of controller design methods that can be used for synchronous chaotic systems (5) and (6); however, these all involve the construction of Lyapunov functions, and some even require the clever design of sliding-mode surfaces, which in turn can be used to invert the synchronous controller u [33]. However, these controller design methods for chaotic systems have specific requirements that are necessary for synchronisation accuracy and setup time. In addition, the fact that these methods require a large number of controllers with a fixed design form makes the practical application of these methods somewhat limited. And the related development of intelligent controllers proves that RBFNN and Fuzzy Neural Networks (FNNs) can approximate the objective function with arbitrary accuracy. Therefore, the design problem of controllers for synchronised chaotic systems (5) and (6) can be transformed into the design problem of suitable parameters for RBFNN controllers. During the design process, these observable parameters should satisfy Equation (7).

(7) $$arg\underset{p\in R^{N_{p}}}{min}\left\{\frac{1}{nN_{x}}\sum_{i=1}^{N_{x}}\sum_{j=1}^{n}(x_{j}^{m}(t_{i})-x_{j}^{s}{\left(u(p),t_{i}\right)}^{2}\right\}$$

where n is the dimension of the state variables of the chaotic system, $N_{x}$ is the length of the numerical solution of the nonlinear system, p is the relevant parameter of the neural network controller that includes c,w,δ, and $N_{p}$ denotes the sum.

### 2.4. Constructed Chaotic System Models

**Figure 2:**
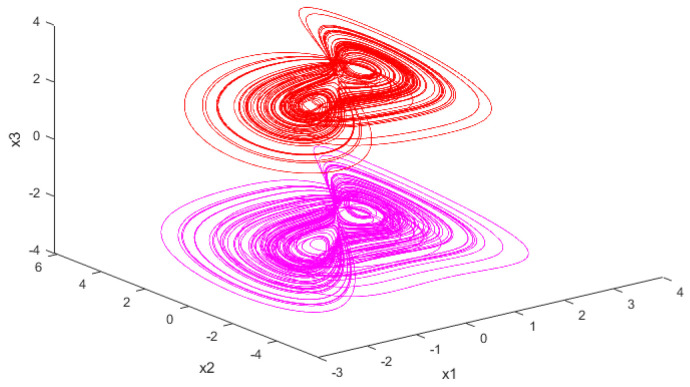
Attractor trajectory diagrams of the Sprott B system corresponding to different initial conditions, with initial condition $x_{0}=[0.1,0.1,1]$ and $x_{1}=[-0.1,-0.1,1]$.

For the purpose of comparison, the equations of the Sprott B chaotic system are chosen as the model of the chaotic system. The expressed equation of Sprott B is written as follows:

(8) $$\left\{\begin{array}{l} {\dot{x}}^{m}=a(y^{m}-x^{m})\\ {\dot{y}}^{m}=bx^{m}z^{m}f(z^{m})\\ {\dot{z}}^{m}=c-x^{m}y^{m} \end{array}\right.$$

where $f(z)=\sum_{i=1}^{n}\left(z-l_{i})(z+l_{i}\right)$, and $l_{i}$ is constant; moreover, $l_{i}=2.5$ is defined, and *n* can take the value of 1, or 2, or 3, or 4, depending on the actual situation. In this paper, *n* takes the value of 1. When a=5,b=2,c=1, given the corresponding initial conditions, the Sprott B system is in a chaotic state and has two chaotic attractors [17]. Figure 2 gives the attractor case for the Sprott B system. In Figure 2, the image depicted by the red line at the top is the phase diagram of the Sprott B system when the initial value is $x_{0}=[0.1,0.1,1]$, and the image depicted by the pink line at the bottom is the phase diagram of the Sprott B system when the initial value is $x_{1}=[-0.1,-0.1,1]$.

**Figure 3:**
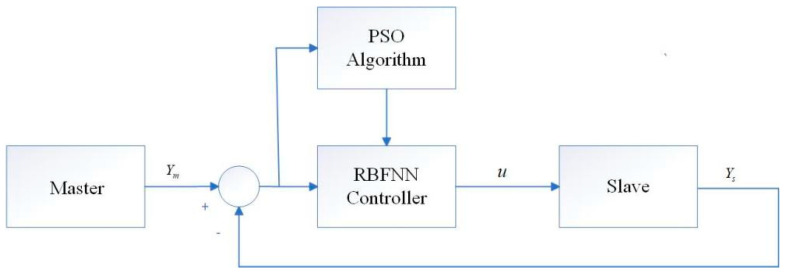
Block diagram of master–slave Sprott B hybrid system synchronisation scheme.

The Sprott B system is used as the basic model of the master system, and the master chaotic system is used in Equation (8), which in turn constructs the slave chaotic system as if it were shown in Equation (9) below.

(9) $$\left\{\begin{array}{l} {\dot{x}}^{s}=a(y^{s}-x^{s})\\ {\dot{y}}^{s}=bx^{s}z^{s}f(z^{s})+g(t)+u(t)\\ {\dot{z}}^{s}=c-x^{s}y^{s} \end{array}\right.$$

where g(t) is used to simulate the extra additional noise of the system, in which $g(t)=2\sin(\pi x^{s})(1+\cos(\pi z^{s})$ is set to facilitate the simulation solution. Let u(t) be the controller of the response system, and according to the RBFNN theory, the Gaussian function is used as the radial basis function to obtain the expression of the controller u(t), which is as follows:

(10) $$\left\{\begin{array}{l} u(t)=w\times \phi (e(t))\\ \phi (e(t))={\left[\phi_{1}(e(t))\;\phi_{2}(e(t))\cdots \phi_{n}(e(t))\right]}^{T}\\ \phi_{i}(e(t))=\exp(-\frac{1}{\delta_{i}^{2}}\sum_{j=1}^{n}{\left(e_{j}(t)-c_{i\cdot j}\right)}^{2})\\ e(t)={\left[e_{1}(t)\;e_{2}(t)\;e_{3}(t)\right]}^{T}={\left[x^{s}-x^{m}\;y^{s}-y^{m}\;z^{s}-z^{m}\right]}^{T} \end{array}\right.$$

where n=3, which is the number of neurons. $w(t)=\left[w_{1}(t)w_{2}(t)\;\cdots w_{n}(t)\;\right]$ is the weight of the radial basis function, and $\delta_{i}$ is the value of the width of the radial basis function $\phi_{i}(t)$. And $c_{i\cdot j}(j=1,2,\cdots ,n)$ is the centre position of the radial basis function $\phi_{i}(t)$. $x^{m},y^{m},z^{m}$ and $x^{s},y^{s},z^{s}$ are the state vectors of the master chaotic system and slave chaotic system, respectively. Moreover, $e(t)=x^{m}-x^{s}$ denotes the state error vector of the master chaotic system and slave chaotic system. Figure 3 illustrates the master–slave system synchronisation scheme.

In order to verify the feasibility of the proposed scheme, the Lorenz chaotic system is selected and compared with the linear feedback synchronization and RBFNN-PSO synchronization schemes, respectively. The Lorenz mixed-degree master system equations are given in Equation (11).

(11) $$\left\{\begin{array}{l} {\dot{x}}_{m}={\;a(y}_{m}{-x}_{m})\\ {\dot{y}}_{m}=rx_{m}-y_{m}-x_{m}y_{m}\\ {\dot{z}}_{m}=x_{m}y_{m}-bz_{m} \end{array}\right.$$

**Figure 4:**
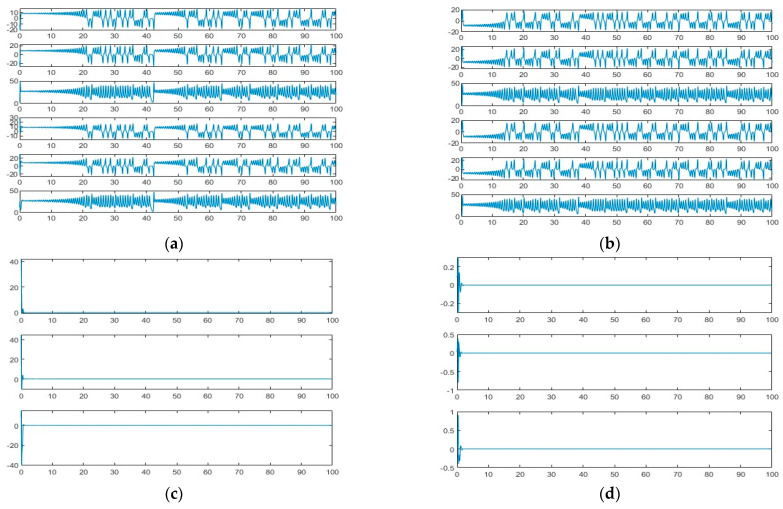

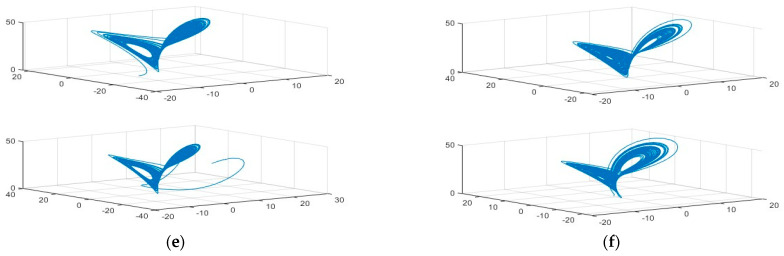
Comparison of simulation results: (**a**) phase diagram of a state variable under a linear feedback synchronization scheme; (**c**) state error of a state variable under a linear feedback synchronization scheme; (**e**) attractor phase diagram of a state variable under a linear feedback synchronization scheme; (**b**) phase diagram of state variables under the RBFNN-PSO synchronization scheme; (**d**) state errors under the RBFNN-PSO synchronization scheme; (**f**) attractor phase diagram under the RBFNN-PSO synchronization scheme.

The equation of the chaotic Lorenz system using linear feedback synchronization is shown in Equation (12).

(12) $$\left\{\begin{array}{l} {\dot{x}}_{s}=a(y_{s}-x_{s})\\ {\dot{y}}_{s}=rx_{s}-y_{s}-x_{s}y_{s}+u\\ {\dot{z}}_{s}=x_{s}y_{s}-bz_{s} \end{array}\right.$$

where $u=K(y_{m}-y_{s})=Ke_{2}(t)$ is the linear feedback controller and K is the feedback gain. The equations of the chaotic Lorenz system using the RBFNN-PSO synchronization scheme are shown in Equation (13).

(13) $$\left\{\begin{array}{l} {\dot{x}}_{s}=a(y_{s}-x_{s})\\ {\dot{y}}_{s}=rx_{s}-y_{s}-x_{s}y_{s}+\sum_{i=0}^{5}w_{i}\Phi_{i}\\ {\dot{z}}_{s}=x_{s}y_{s}-bz_{s} \end{array}\right.$$

where $u=\sum_{i=0}^{5}w_{i}\Phi_{i}$ is the RBFNN controller, $\Phi_{i}$ is the mirror base Gaussian function and $w_{i}$ is its weights. Numerical simulation is carried out using MATLAB 2022B and the results are shown in Figure 4. From an analysis of Figure 4, the synchronization results obtained by using the chaotic Lorenz system with linear feedback synchronization and the RBFNN-PSO synchronization scheme, respectively, are close to each other, which proves that the proposed RBFNN-PSO synchronization scheme is feasible.

## 3. Synchronisation Schemes for Master–Slave Sprott B Chaotic Systems with Additional Noise

### 3.1. Training Process for PSO Parameters

In this section, considering Equation (8) as the master chaotic system and Equation (9) as the slave chaotic system, the RBFNN controller is directly involved in the mediation process of the response system and is responsible for performing the role of the controller. The Advanced Particle Swarm Optimisation algorithm (APSO) is indirectly involved in the process rectifying the parameters of the response system, which is responsible for finding the optimal values of the parameters of the neural controllers through pre-training. At the same time, the main process of synchronising Equations (8) and (9) is transformed into determining the appropriate parameters of the neural network and system coefficients that will be used as controllers.

**Figure 5:**
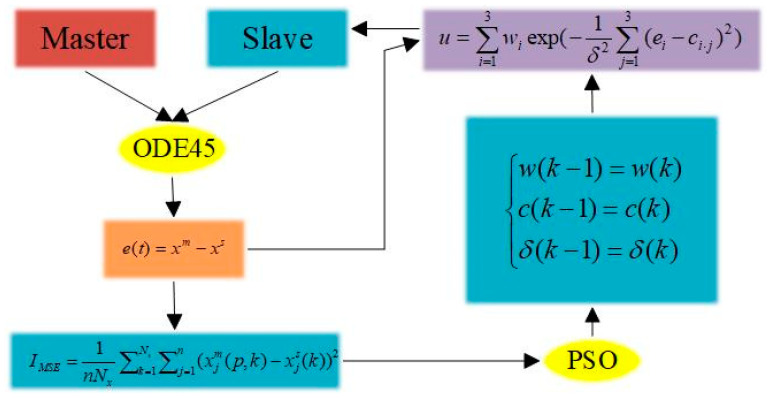
Block diagram of the training scheme for the overall RBFNN-PSO algorithm.

According to the requirements of the PSO optimisation algorithm, the fitness function must be pre-designed before the introduction of the optimisation controller. And the fitness function must be effective and direct, which should reflect the degree of approximation of the objective function under the current conditions and simplify the whole operation process as much as possible to improve the computational efficiency. In order to overcome the drawbacks of computational complexity and the high cost of integral mean error (IEA), the mean square error (MSE) is chosen here as the fitness function to reduce the computational complexity. The equation for fitness is specified in Equation (14).

(14) $$I_{MSE}=\frac{1}{nN_{x}}\sum_{k=1}^{N_{x}}\sum_{j=1}^{n}(x_{j}^{m}(p,k)-x_{j}^{s}(k){)}^{2}$$

where $N_{x}$ is the length of the numerical solution of the chaotic system during the training process, and $x^{m}(p,k)\in R^{n}$ is the state of the main chaotic system at time *k*. $x^{s}(k)\in R^{n}$ is the state of the slave chaotic system at time *k*; additionally, $p={\left[w^{T}c^{T}\delta^{T}\right]}^{T}\in R^{N_{p}}$ is the parameter that the RBF controller is waiting to learn. The parameters w,c,δ are stored sequentially in parameters N, n×N and N, respectively. Note that $N_{p}=N\times (2+n)$. Figure 5 illustrates the training scheme of the overall RBFNN-PSO algorithm.

The steps for training the parameters of the PSO algorithm are as follows:

**Step 1:** Determine the elements of the particle vector X, including the three controller training parameters w, c and δ, to form the 25 × 1 dimensional matrix that is shown in Equation (15); then, randomly assign the initial values of the parameters $w_{0},\;c_{0}$ and $\delta_{0}$;

(15) $$X={\left[w_{1}\;w_{2}\;w_{3}\;w_{4}\;w_{5}\;c_{1}\;c_{2}\;c_{3}\cdots c_{15}\delta_{1}\;\delta_{2}\;\delta_{3}\;\delta_{4}\;\delta_{5}\right]}^{T}$$

**Step 2:** Solve the master–slave chaotic system equations in a certain interval by using the MATLAB library function ODE45;

**Step 3:** Quantitatively evaluate the performance of the parameters using the fitness function ($I_{MSE}$);

**Step 4:** Based on the above evaluation, select the automatic update parameters of the PSO optimisation algorithm;

**Step 5:** Repeat steps 2–4 until the minimisation of the value of the fitness function or the maximum number of search iterations defined by the algorithm is reached;

**Step 6:** Fix the controller parameters of the RBFNN algorithm and the parameters of the system.

The pseudo-code for the calculation of the fitness function and the pseudo-code for the PSO parameter training process are given in Table 1 and Table 2, respectively.

The value of the fitness function is the key condition for the parameter training of the PSO algorithm, and the solution of the master–slave chaotic system and the corresponding time are thus given by the ODE45 library function in MATLAB. Then, the solution of the master–slave chaotic system is subjected to the mean square error operation and finally $I_{MSE}$ is obtained. The functions and methods described above provide us with the ability to create an optimal neural controller for the slave chaotic system. The controller is capable of generating the same motion and chaotic sequences as the master chaotic system, while maintaining a specified level of accuracy. After these several processing steps, we obtained two different chaotic sequences, called $Y_{m}$ and $Y_{s}$, to facilitate the subsequent application of the synchronised system.

### 3.2. Synchronisation Characterisation of Chaotic Systems

**Figure 6:**
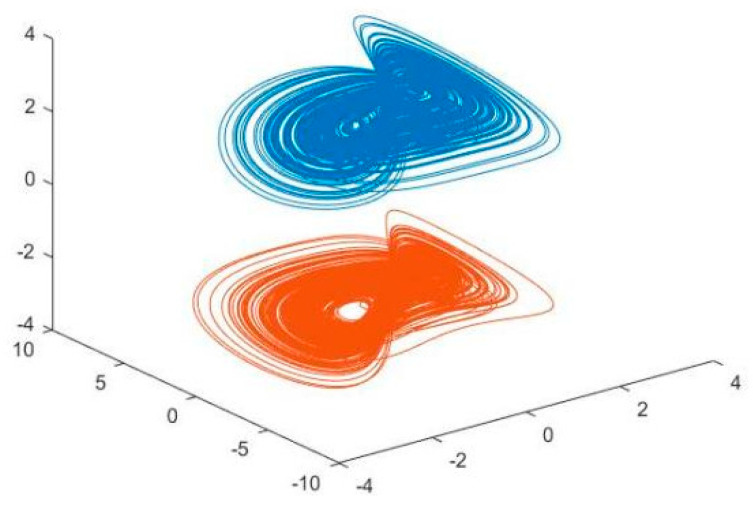
Trajectory diagram of the Sprott B master–slave chaotic system trained by PSO.

**Figure 7:**
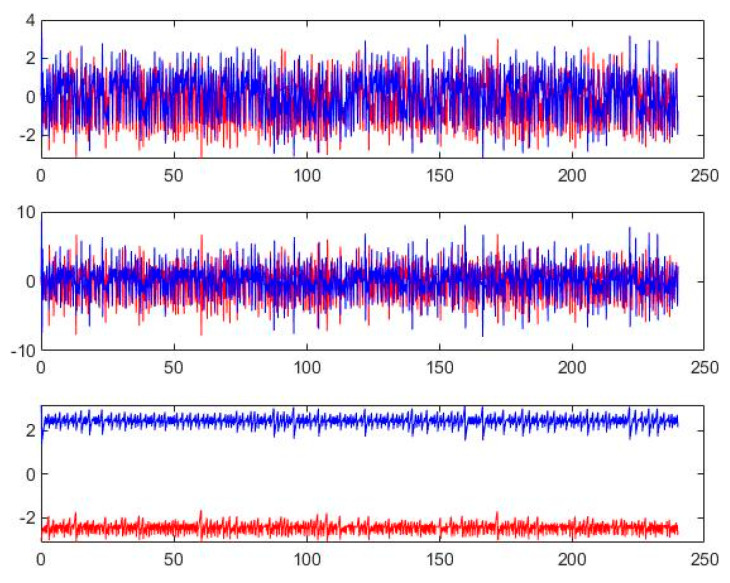
State trajectory of the Sprott B master–slave system after being trained by the PSO algorithm.

In this section, Equation (8) is used as the master chaotic system and Equation (9) is used as the slave chaotic system, with the initial values chosen as A and B, respectively. The RBFNN algorithm is used to set up the controllers in accordance with Equation (10) and the controllers are given the corresponding initial values. Thereafter, the number of particles for the PSO algorithm is set to 1000 while the number of iterations is set to 50, followed by the training of the controller parameters. The final trajectory diagrams of the master Sprott B system and the slave Sprott B system, as well as the trajectory diagrams of each state variable, are shown in Figure 6 and Figure 7, respectively. In Figure 6, the image depicted by the blue line at the top is the phase diagram of the Sprott B system when the initial value is $x_{0}=[0.1,0.1,1]$, and the image depicted by the orange line at the bottom is the phase diagram of the Sprott B system when the initial value is $x_{1}=[-0.1,-0.1,1]$.

From the state trajectories of the Sprott B master–slave system trained by the PSO algorithm in Figure 7, the blue and orange iteration curves correspond to the blue and orange systems in Figure 6, respectively, andwhere the master system is in blue and the slave system is in red. It can be seen that the Sprott B master–slave chaotic system trained by PSO using the RBFNN controller retains the chaotic properties of the source system, and has a good sensitivity to the initial value of different initial conditions.

## 4. Application of the Introduced Synchronization Scheme in Image Encryption System

### 4.1. Proposed Image Encryption Scheme

**Figure 8:**
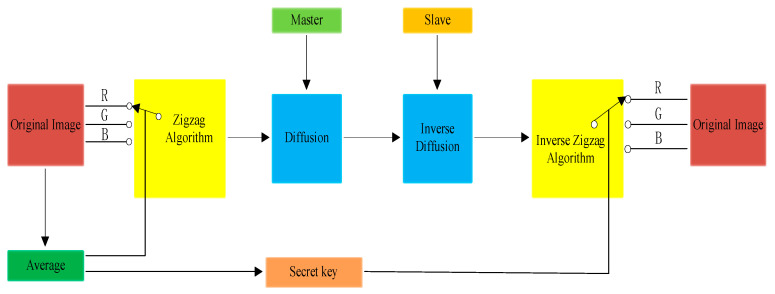
Block diagram of image encryption scheme.

In most of the existing research on image encryption, the initial values of the chaotic system are used as the initial key; therefore, if the initial key is lost, although the image encryption system can rely on the interrupt algorithm alone for encryption, this greatly reduces the security of the algorithm. Accordingly, this paper implements image encryption based on the chaotic synchronisation method, which overcomes the disadvantage of relying on the initial value of the chaotic system for encryption. An enhanced zigzag destruction and diffusion method is proposed to solve the problem of the low periodicity of the initial transformation of the image, and the complete image encryption scheme is given in Figure 8.

### 4.2. Zigzag Image Scrambling Scheme

The image scrambling algorithm is an information-hiding technique that enables the hiding and protection of image information as the image after the scrambling algorithm is unrecognisable. The main purpose of image scrambling is to turn a given image into a random image without explicit information after scrambling. According to the characteristics of image scrambling, image scrambling can be divided into space-domain scrambling, frequency-domain scrambling, and mixed space–frequency-domain scrambling, so this paper considers image scrambling in the space-domain.

**Figure 9:**
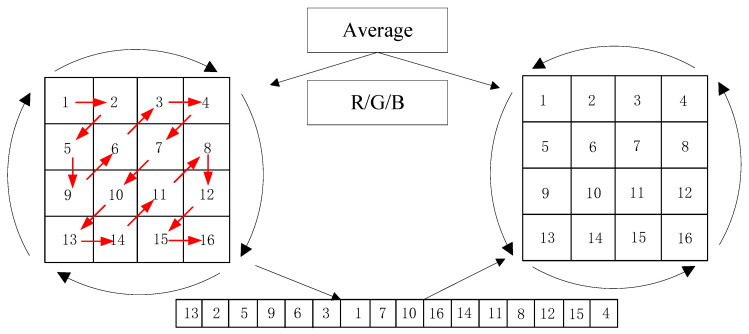
Schematic diagram of enhanced Zigzag disambiguation algorithm (4 × 4 RGB image).

To enhance the complexity of Zigzag image disarrangement, an enhanced disarrangement algorithm is proposed, as shown in Figure 9. The Zigzag image scrambling algorithm is a scrambling technique in the spatial domain of an image, which rearranges the pixels of an image through the Zigzag scanning method in order to encrypt or protect the image information. This technique is not only applicable to grey-scale images, but also to colour images, which improves the security and confidentiality of images by causing double disruption in the spatial and colour domains.

As can be seen from Figure 9, where the red arrow indicates the direction of movement, the enhanced Zigzag algorithm firstly adopts this way of exchanging the top corner positions with each other, which overcomes the problem of the first and the last data positions remaining unchanged in the Zigzag scrambling algorithm due to easy cracking; and secondly, it adopts the order of the size of the mean value of the original image to disrupt the pixel values of the R, G, and B channels, respectively, which enhances the complexity of the image disarrangement. The mean value of the image is sent to the receiver as the initial key to be used in image decryption. Finally, the scrambled data stream is generated after scrambling by the enhanced Zigzag algorithm.

### 4.3. Image Diffusion Program

The diffusion algorithm involves the process of dispersing an input message into multiple parts that can ultimately reduce the risk of the plaintext message being deciphered. In cryptography, diffusion algorithms usually refer to the dispersal of a plaintext message into multiple ciphertext messages, thereby reducing the likelihood of an attacker gaining access to the complete message.

The encryption process is as follows: take sequences $Y_{m}$, $Y_{s}$ and $Y_{m}$, which were generated by the chaotic synchronisation system in Section 3.1, and use them as the sequences of the diffusion algorithm part of the encryption system, which in turn performs a heterodyne operation with the disrupted data stream $P_{m}$ to form the encrypted data stream $E_{m}$ ($E_{m}=P_{m}⊕Y_{m}$). The specific encryption formula is as follows:

(16) $$\left\{\begin{array}{l} Y_{m}=mod(Y_{m}\times 10^{6},256)\\ E_{m}=P_{m}⊕Y_{m} \end{array}\right.$$

The decryption process is as follows: the encrypted data stream $E_{m}$ is received by the receiver through the channel, and the receiver uses the synchronisation sequences $Y_{s}$ and $E_{m}$, which are generated by the chaotic system, and performs the heterodyne operation with the data streams $Y_{s}$ and $E_{m}$ to obtain the disrupted data stream $D_{s}$ ($D_{s}=E_{m}⊕Y_{s}$). The Zigzag inverse scanning operation is then implemented to recover the original image. The specific decryption formula is as follows:

(17) $$\left\{\begin{array}{l} Y_{s}=mod(Y_{s}\times 10^{6},256)\\ D_{s}=E_{m}⊕Y_{s} \end{array}\right.$$

### 4.4. Simulation Results of the Encryption System

#### 4.4.1. Analysis of Encryption and Decryption Results

**Figure 10:**
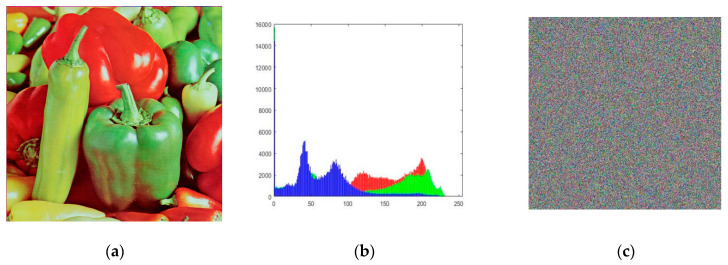

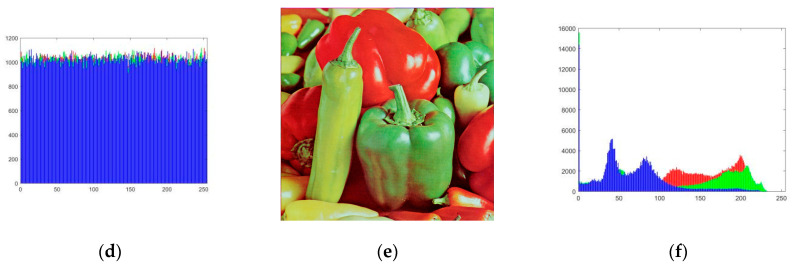
Encryption and decryption performance based on Sprott B master–slave chaotic system: (**a**) plaintext image; (**b**) histogram of the R, G, and B channels of the plaintext data; (**c**) ciphertext image; (**d**) histogram of the R, G, and B channels of the ciphertext data; (**e**) decrypted image; (**f**) histogram of the R, G, and B channels of the decrypted image data.

Adopting the enhanced Zigzag image disambiguation method introduced in this paper, the chaotic sequence generated by the master system of Sprott B is used to operate the diffusion algorithm on the disambiguated image during the encryption process, and then the received encrypted image is decrypted by the chaotic sequences generated by the synchronised Sprott B slave system; the results are shown in Figure 10.

#### 4.4.2. Histogram Analysis

In order to analyse this more intuitively, this study carried out a histogram analysis of the original image, the encrypted image, and the decrypted image, and it can be seen that the encryption and decryption have better results. In this paper, the histogram method is used to evaluate the uniformity of the pixels of the encrypted image and the statistical value $\chi^{2}$ is defined as follows:

(18) $$\chi^{2}=\sum_{i=0}^{255}\frac{{\left(E_{i}-Y\right)}^{2}}{Y}$$

where $E_{i}$ is the current pixel value and Y is the frequency of occurrence expected for each pixel. When the $\chi^{2}$ value of the cipher image is calculated to be not more than 293.2478, the encrypted image can be used according to the method of chi-square evaluation [34] (Table 3).

**Figure 11:**
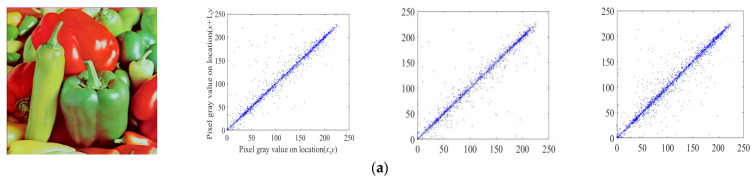

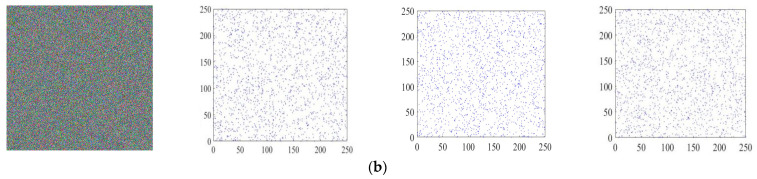
The correlation distributions: (**a**) the plaintext image and correlation distributions of three directions; (**b**) the ciphertext image and correlation distributions of three directions.

The chi-square value of each type of encrypted image is shown in Figure 11, and it can be seen that the chi-square value of each encrypted image is not more than 293.2478; therefore, the distribution of the pixel values of the encrypted image is uniform.

#### 4.4.3. Shannon Entropy of Encrypted Images

In this paper, Shannon entropy is used to evaluate the random distribution of pixels in an encrypted image, and Shannon entropy is defined as follows:

(19) $$H(R)=-\sum_{i=0}^{N-1}P(R_{i})\log_{2}P(R_{i})$$

where N and R are the maximum and individual pixel values of the image, respectively. P(⋅) is the discrete probability density function. Table 4 lists the Shannon entropy of some of the test images, and it can be seen that the average value of the Shannon entropy of the encrypted image is 7.9937, which is closer to 8, indicating that the encrypted image is uniformly distributed.

#### 4.4.4. Correlation Analysis

The pixel correlation of an image covers three directions: horizontal, vertical, and diagonal. Thus, for a good encryption algorithm, the goal should be to reduce the correlation between adjacent pixels. This can be defined as the correlation between two pixel sequences, which is given as follows:

(20) $$r_{uv}=\frac{\mathrm{cov}(u,v)}{\sqrt{D(u)}\sqrt{D(v)}}$$

(21) $$\mathrm{cov}(u,v)=\frac{1}{N}\sum_{i=1}^{N}\left(u_{i}-E(u))(v_{i}-E(v)\right)$$

(22) $$E(u)=\frac{1}{N}\sum_{i=1}^{N}u_{i}$$

where u and v are adjacent pixels values, and $r_{uv}$ is the correlation coefficient of the adjacent pixels. The 4500 pairs of adjacent pixels—from the plaintext and encrypted images in the horizontal, vertical, and diagonal directions—are randomly selected. The distribution of the 4500 pairs is shown in Figure 11. The plaintext image has pixels close to the diagonal, while the ciphertext image has a random distribution of pixels, which can be seen in Figure 11. The comparative results of the correlation obtained by using different encryption schemes are presented in Table 5.

#### 4.4.5. Differential Attack

When little modifications to the source picture cause substantial changes to the encrypted image, differential attacks are largely ineffective. Number of pixels change rate (NPCR) and unitary averaged changed intensity (UACI) tests are used to assess the capacity of the proposed picture encryption methods to withstand differential assaults. The NPCR and UACI can be expressed as follows:

(23) $$NPCR=\frac{\sum_{m=1}^{M}\sum_{n=1}^{N}D(m,n)}{MN}\times 100\%$$

(24) $$D(m,n)=\left\{\begin{array}{l} 1,\;\;for\;C_{1}(m,n)\neq C_{2}(m,n);\\ 0,\;\;\;\;otherwise. \end{array}\right.$$

(25) $$UACI(C_{1},C_{2})=\sum_{m=1}^{M}\sum_{n=1}^{M}\frac{\left|C_{1}(m,n)-C_{2}(m,n)\right|}{256\times M\times N}\times 100\%.$$

where $C_{1}$ and $C_{2}$ denote the two encrypted pictures, which are identical to the original images except for a single additional pixel, and D(m,n) is the total number of pixels in the encrypted images $C_{1}$ and $C_{2}$. The ideal expected NPCR and UACI values are 99.61% and 33.46%, respectively. The suggested encryption scheme’s mean NPCR and UACI values are shown in Table 6. These findings show that the suggested method performs better in terms of defending against differential assaults.

## 5. Conclusions

In this paper, to address design inflexibility problems such as the chaotic system synchronisation design relying on specialised controllers and the lack of chaotic systems with uncertain parameters and external noise, the RBFNN-PSO chaotic system design methodology is proposed, and the RBF neural network controller is designed, with the PSO training scheme given to achieve the synchronisation of two chaotic systems. The subsequent simulation results show that the designed controller has better robustness to noise. In addition, the designed chaotic synchronisation system is applied to the image encryption system with the enhanced Zigzag disruption method, and the final simulation results show that the chaotic system encryption has better confidentiality and attack resistance.

## Figures and Tables

**Table 1:** The pseudo-code for the calculation of the fitness function.

**Input:** w,c,δ**,**tspan**, *y*_0_;** **Output: *I_MSE_*;**
initialize $w=w_{0},c=c_{0},\delta =\delta_{0}$, a,b,d=[5,2,1];
[*t*, *y_m_*] = ODE45 (Sprott B (a,b,d), *tpan*, *y*_0_); [*t*, *y_s_*] = ODE45 (Sprott B (w,c,δ,a,b,d), *tpan*, *y*_0_);
*L* = length(*t*);
initialize *I_MSE_* = 0;
for *k*: 1 to *L*
$I_{MSE}=I_{MSE}+\sum_{i=1}^{n}(y_{i}^{m}(k)-y_{i}^{s}(k){)}^{2}$
end for
*I_MSE_* = *I_MSE_*/(n*L);

**Table 2:** The pseudo-code for the training process of the parameters of the PSO algorithm.

**Input: F_P_;** Output: p*;
intonation: *N* = 5, *N_p_* = 5*N, *num_particles* = 1000, *num_iteration* = 50, *r*_p_ = *r*_q_ = 2, *wl* = 0.3, *wu = 0.95*, *y*_0_ = [*y*_01_,*y*_02_], *xpan*, *lb* = −125, *ub* = 125, *p_position,g_position*ϵR^5*N^, *cost*, *v_p_*, *v_g_* = inf;
generate randomly: *p* = *p_position* = *g_position* = *p_0_*;
for i = 1 to *num_particles*;
*cost*(i) = F_P_(*p*(i), *y*_0_, *xpan*);
update *v_p_* and *v_g_* according to comparative result with cost;
end for
for ite = 1 to *num_iteration_t_*;
*w*_1_ = *w*_b_(2) − (*w*_b_(2) − *w*_b_(1)) * ite/*num_iteration*;
for i = 1 to *num_particles*;
*q*(i) = *w*_1_ * *q*(i − 1) + *c_p_* * *r*_p_ * (*p_p_* − *p*(i − 1)) + *c_q_* * *r*_q_ * (*p_q_* − *p*(i − 1));
restric the scope of *q*;
*p*(i) = *q*(i); restic the scope of *p*;
*cost*(i) = F_P_(*p*(i), *y*_0_, *xpan*);
update *v_p_* and *v_g_* according to comparative result with cost or the cost small enough;
end for
end for

**Table 3:** The $\chi^{2}$ value of the encrypted image.

Images	Lena	Ruler	Gray	Pepper	Boat
$\chi^{2}$	247.985	242.063	237.382	217.067	245.313

**Table 4:** Shannon entropy values of some original and encrypted images.

File Name	Original Image	Encrypted Image
**Lena**	7.7319	7.9920
**Ruler**	0.5000	7.9956
**Gray**	4.3923	7.9970
**Pepper**	7.6698	7.9920
**Boat**	7.1941	7.9833
**Mean value**	5.4973	7.9920

**Table 5:** Adjacent pixel correlations of the plaintext image “Pepper” and the proposed method for the ciphertext image.

Schemes	Horizontal	Vertical	Diagonal
“Pepper” image	0.9831	0.9835	0.9723
Proposed method	−0.00100	−0.00093	0.00100

**Table 6:** The NPCR and UACI values of the ciphered images.

Images	NPCR (%)			UACI (%)		
	R	G	B	R	G	B
Pepper	99.37	99.62	99.58	33.42	33.37	33.41

## Data Availability

The data presented in this study are available on request from the corresponding author.
